# The synapse: people, words and connections

**DOI:** 10.1042/NS20220017

**Published:** 2022-06-08

**Authors:** E. M. Tansey

**Affiliations:** William Harvey Research Institute, Barts and The London, QMUL U.K. and Neuroscience, Physiology, and Pharmacology, UCL, U.K.

**Keywords:** Dale,Henry, history of neuroscience, neurotransmission, Sherrington,Charles, synapse

## Abstract

This paper provides a review of some of the major historical developments in synaptic research and neurotransmission since the first appearance of the word ‘synapsis’ in 1895. The key contributions and inter-relationships of several significant scientists and Nobel Laureates, including Charles Sherrington, Henry Dale, Edgar Adrian and John Eccles are highlighted, and the influence of others such as John Langley and Thomas Elliott is stressed. A recurrent theme is the importance of language and the creation of new words.

“When I use a word”, said Humpty Dumpty, “it means just what I choose it to mean, neither more nor less” [1]. Charles Sherrington, the Professor of Physiology at Liverpool, was clearly of a broadly similar opinion, as he declared in a letter in 1897 to his colleague Edward Schäfer, Professor of Physiology in Edinburgh:

“As to nomenclature—its sole object is I take it clearness combined with brevity … Definition is wanting when a penny has to pass for 5 & 3 farthings as well as for 4: the one symbol is then too little … All I think we ought to be careful not to do is ‘committing barbarisms,’ e.g., impossible adjectival form, using prefixes and affixes with false signification or in impossible ways—that simply adds new terms which like other ‘monsters' can’t live long, and may be misleading during life, does all of us harm as giving impression of carelessness or ignorance” [2].

The correspondence had been prompted by the appearance of the word ‘synapsis’ (subsequently modified to ‘synapse’) in the chapter on ‘The Spinal Cord’ in the seventh edition of Michael Foster’s *Textbook of Physiology.* The author had carefully explained its meaning:

“So far as our present knowledge goes we are led to think that the tip of a twig of the [axon's] arborescence is not continuous with but merely in contact with the substance of the dendrite or cell body on which it impinges. Such a special connection of one nerve cell with another might be called a synapsis”

presciently suggesting a useful hypothesis,

“the lack of continuity between the material of the arborisation of the one cell and that of the dendrite (or body) of the other cell offers an opportunity for some change in the nature of the nervous impulse as it passes from one cell to the other” [3].

But who was that author?

## Michael Foster (1836–1907), Charles Sherrington (1857–1952) and Edward Schäfer (1850–1935)

**Figure 1:**
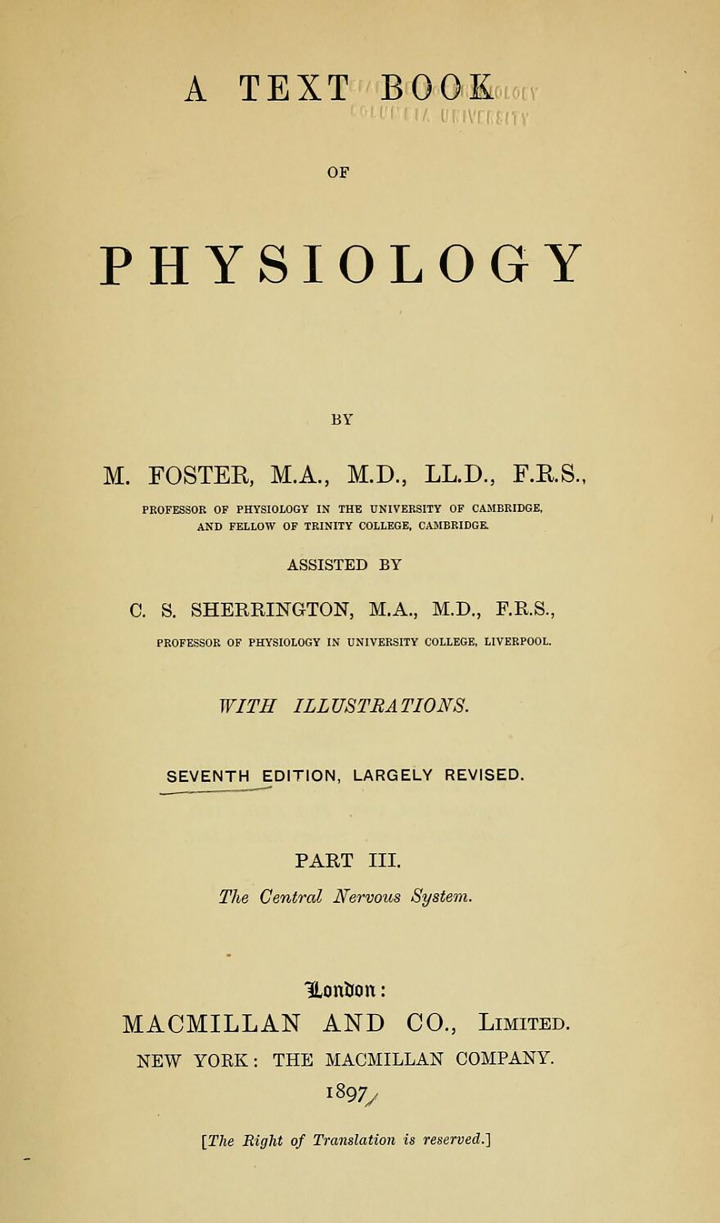
Title page of the third volume of Michael Foster (1897) ‘*A Textbook of physiology*’ in which the word ‘synapse’ first appeared (source Wellcome Images)

Understandably, the word is often attributed to Michael Foster, the Professor of Physiology at Cambridge, and the doyen of British physiology [4]. Nevertheless, it can be clearly demonstrated that it is to Charles Sherrington, Foster’s former pupil, that much credit should be given, for not only creating the word itself but also for advocating the concept of a synapse, most notably from his work on physiological reflexes. In the Foreword to the volume on the nervous system in that edition of his *Textbook*, Foster generously acknowledged, “My friend, Professor Sherrington, has given me throughout such large help that I have thought it due to him that his name should appear with mine on the title page” [5]. However, Sherrington is only credited with providing ‘assistance’ and no distinction is made between the two men’s contributions in the text itself (Figure 1). By consulting the correspondence of Foster, Sherrington and others, a clearer picture emerges. At the same time as Foster’s *Textbook* appeared, Edward Schäfer was planning his own, more extensive, multi-authored *Textbook of Physiology* to which Sherrington was invited to contribute four chapters on elements of the nervous system [6]. Schäfer was slightly uneasy with the new word and proposed that ‘junction’ might suffice. For Sherrington however, that word was too inflexible and suggested a physical union, redolent of the reticulum, rather than the neuron, theory (see below):

“The mere fact that junction implies passive union is alone enough to ruin the term … I think it does not want the gift of prophecy to foretell that it [junction] must become more & more obviously inapplicable as research progresses. Synapse, which implies a catching on, as e.g., by one wrestler of another—is really much closer to the mark. But I am not a bit wedded to the word: if you could suggest an English word containing the notion which is not already overburdened with applications. I have been trying to find one but cannot. Conjunction is even worse than junction” [7].

So where did the word ‘synapse’ come from? Sherrington in his correspondence with Schäfer admits “Synapsis is none of my making, it comes from a well-known ‘Grecian’ and metaphysician after confabulation with Foster” [2]. A much later letter, in 1937, from Sherrington to the Professor of Physiology at Yale, John Fulton (1899-1960), reveals the identity of Foster’s colleague. Sherrington described how he started revising Foster’s *Textbook* and recalled,

“[I] had not got far before I felt the need of some name to call the junction between nerve-cell and nerve-cell … I wrote him [Foster] of my difficulty, and my wish to introduce a specific name. I suggested using syndesm (σύνδϵσμος). He consulted his Trinity friend Arthur Woolgar Verrall, the Euripidean scholar and [later] professor of English at Trinity College, and Verrall suggested ‘synapse’ (from συνάπτω) and as that yields a better adjectival form, it was adopted for the book… ‘Synapsis’ strictly means a process of contact—i.e. a proceeding or act of contact, rather than a thing which enables contact i.e. an instrument of contact” [8].

Throughout the exchanges Sherrington took immense trouble to emphasise that the word must encapsulate the essentially *dynamic* process that he envisaged. He considered the Latin “jungere”, suggesting a yoking together or a physical joining and the Greek “σύνδϵσμος” meaning binding together, too permanent and passive for his liking, but “capere” to seize, and “συνάπτω” expressing an active “touching together” were more suitable [9]. Sherrington’s concept is clearly recognizable today, and his insistence on finding an appropriate, unequivocal word has been of major importance in twentieth century neuroscience, although the credit for the word itself belongs not solely to the neurophysiologist and should be shared with a Cambridge classicist [10].

### Structural and functional units: the neuron: Camillo Golgi (1843–1926) and Santiago Ramón y Cajal (1852–1934); the reflex arc: Charles Sherrington (1857–1952); and frequency code: Edgar Adrian (1889–1977)

Physiologists were not alone in struggling to understand the structure and function of the nervous system, and especially that of neural connectivity [11]. Anatomists too were deeply involved in debates about neural structure, the two main protagonists being Camillo Golgi, an Italian anatomist who postulated that the nervous system was a continuum, a net-like reticulum; whilst the Spaniard Santiago Ramón y Cajal, convincingly using innovative histological techniques developed by Golgi, proposed that the nervous system was discontinuous, comprising a large number of individual specialised nerve cells or neurons. By the end of the nineteenth century the views of Cajal were gaining ascendancy over those of Golgi, although the two men, each firmly wedded to their opposing views, shared the Nobel Prize in Physiology or Medicine in 1906. The widespread acceptance of Cajal’s neuron theory has provided one of the cornerstones of modern neuroscience. Sherrington and his colleagues, from their work on reflex actions in particular, provided much information about the possible functional nature of the synapse, but until the advent of the electron microscope a similarly detailed morphological basis for thinking about and investigating the synapse was absent. The experimental investigations of the mechanism by which the presynaptic nerve impulse evokes further post synaptic effects, by Dale and others in the first half of the twentieth century, took place therefore almost entirely within the context of debates and disputes about neuronal function [12].

Cajal's advocacy of the neuron as the anatomical unit of the nervous system deeply influenced Sherrington, with whom the Spaniard stayed when visiting the UK in 1894 to deliver the Croonian Lecture to the Royal Society [13–15]. Sherrington’s own neurophysiological work led him to propose the ‘reflex arc’ as the analogous, functional, unit of the nervous system, the simplest arc being comprised just an input and an output neuron with a synapse between them. He further showed that complex activities such as walking could be broken down into a series of component reflex arcs at successively higher levels, and he advanced the phrase ‘the final common pathway’ for the confluence of reflex arcs from many different sensory inputs onto one output neuron which innervated the effector muscle [16]. It is pertinent to remember that the first clear electrical recordings from the nervous system were not made until the 1920s when, amongst others, Alexander Forbes, Edgar Adrian, Herbert Gasser and Joseph Erlanger all provided compelling evidence of action potentials [17]. Like Sherrington, Adrian was a product of the Cambridge Physiological Laboratory, his principal influence there being the neurophysiologist Keith Lucas (1879–1916). Adrian analysed activity in sensory and motor nerves, and developed a particularly sensitive technique to record action potentials from single, isolated nerve fibres [18]. He not only formulated the ‘all-or-none’ law of nerve action but also recognised that information was conveyed along the nerve fibre by variations in the frequency, which he termed the ‘frequency code’ of neural functioning [19].

However, what happened at the synapse?

## Ganglionic transmission: John Langley (1852–1925)

An important contribution to the elucidation of synaptic functioning came from the work of another Cambridge physiologist of an older generation, J. N. Langley, who studied components of what was then variously called the ‘organic’, ‘vegetative’, ‘ganglionic’, ‘visceral’ or ‘involuntary’ nervous system. Langley, in one of his own chapters in Schäfer’s *Textbook*, proposed “following a suggestion of Professor Jebb, to use the word ‘autonomic’, including under the term the contractile cells, unstriated muscle, cardiac muscle, and gland cells of the body, together with the nerve cells and fibres in connection with them” [20]. Sir Richard Jebb was then Regius Professor of Greek at Cambridge, and not surprisingly, a colleague of Verrall who had advised Sherrington and Foster about ‘synapsis’ [21].

Langley and several of his colleagues and students, including Sherrington, Elliott, Dale, and Adrian, contributed enormously to understanding neural mechanisms. Of particular significance are the experiments in which he investigated functional aspects of the autonomic nervous system by applying chemicals such as nicotine, curare and picrotoxin by painstakingly painting the ganglia with them. He found that nicotine could selectively interrupt the transmission of nerve impulses from the pre-ganglionic to the post-ganglionic nerve fibre in sympathetic ganglia, and fibres ending in the ganglion could be distinguished from those that passed through it [22]. Further experiments indicated that some substances, most notably suprarenal gland extract (now called adrenaline [23]) had a direct action on the effector cells, i.e. the muscle cells and gland cells, rather than nerve endings.

Langley speculated that these reactions depended “upon the presence of a special chemical substance in the nerve-cells or on the nerve-endings” and used the expression ‘receptive substances’ to explain the chemical interactions he observed [24]. Significantly there are obvious parallels with Emil Fischer’s contemporary explanations about enzyme activity as ‘lock-and-key’ actions [25] and Paul Ehrlich’s ‘side chain theory’ to explain toxin/antitoxin interactions in the emerging discipline of immunology [26].

### Towards chemical neurotransmission: Thomas Elliott (1877–1961) and Henry Dale (1875–1968)

It was a student of Langley’s, T R Elliott, who contributed the next piece to the jigsaw of synaptic neurotransmission. Repeating and extending some of Langley’s experiments, he showed quite clearly that in many situations the external application of adrenaline closely paralleled the effects of endogenous stimulation of the sympathetic nerves. Elliott’s very first results, showing that the effects of stimulating the hypogastric nerve could be mimicked by the application of adrenaline, were communicated to The Physiological Society in May 1904. The final sentence of his published abstract reads “Adrenaline might then be the substance liberated when the nervous stimulus reaches the periphery” [27]. The precise meaning or intention of these words is unclear, although they are frequently interpreted as suggesting the release of adrenaline from the nerve ending in response to the passage of a nervous impulse, thus heralding the concept of chemical neurotransmission. At the time however the phrase attracted little serious attention. Quite what Elliott meant by this ambiguous statement is unknown, although it has predominantly been interpreted as the first definitive proposal of chemical neurotransmission, as in a comment over 40 years later, from Dale to E. D. Adrian:

“I quite clearly identify Elliott as the originator of the fundamental idea, which so many years later led to wider developments in the hands of Loewi, on the one hand, and my own group on the other” [28].

Elliott's major paper on the subject, published in the *Journal of Physiology* the following year provided a detailed analysis of the effects of adrenaline on a wide variety of tissues and re-iterated its close correlation to sympathetic stimulation, but offered no further speculation [29]. An important concept he promoted was that of the myoneural junction, an area clearly associated with the post-synaptic muscle cell and not with the pre-synaptic nerve cell, sensitive and responsive to a chemical stimulant. In a more general paper, which he published in the *British Medical Journal* in the same year, he did offer some further elucidation,

“This mechanism, the myoneural junction, serves to *transmit* [my emphasis] the impulse to the muscle, and probably determines whether the response of the latter shall be that of inhibition or contraction….This characteristic reaction to adrenalin marks the deep distinction between the myoneural junctions of the sympathetic (thoracico-lumbar) nerves on the one side, and all the remaining visceral nerves on the other” [30].

Significantly, he still made no speculation of the source of the adrenaline, except “stored… in the neighbourhood of the myoneural junction”, although emphasising that adrenaline imitated only the effects of the sympathetic, but not the parasympathetic, nervous system.

Elliott effectively left physiological research shortly after writing his extensive paper on the physiological actions of adrenaline-he completed medical qualifications, ultimately becoming Professor of Medicine at UCL, and devoting himself to a career in clinical practice and research. His work at Cambridge had been supported by the George Henry Lewes (GHL) Studentship, a rare scholarship to promote physiological research created by the novelist Mary Ann Evans (George Eliot) in memory of her partner [31]. Elliott’s predecessor both as Langley’s student and as GHL student had been Henry Dale who after leaving Cambridge had also finished his medical training at St Bartholomew’s Hospital, and then accepted a unique position as research pharmacologist with the pharmaceutical manufacturer Henry Wellcome in a pioneer institution, The Wellcome Physiological Research Laboratories (WPRL). At Wellcome’s bidding, Dale examined the physiology and pharmacology of ergot of rye, then marketed for use in obstetrics. He showed that using a specific ergot extract, chrysotoxin, in exactly the same experimental conditions that Elliott had used, completely reversed the effects of the applied adrenaline. Additionally, chrysotoxin could reverse the effect of normal sympathetic nerve stimulation [32]. These two separate phenomena were closely related: the effect of applied adrenaline and its closeness to the action of normal sympathetic neural stimulation; and secondly, the apparent ‘reversal’ of those effects by a particular extract of ergot. Both observations were of considerable significance in the development of theories about the transmitter functions of chemicals at synapses in the nervous system.

Dale, inspired and intrigued by Elliott’s experiments, and with the extensive resources of the WPRL at his command (and no teaching or clinical duties), continued to investigate the relationships between chemical structure and physiological actions, especially in relation to functional mimicry of neural activity. He started a major study of the physiological actions of 56 chemicals structurally similar to adrenaline, many specially synthesised by his chemist colleague George Barger, by examining their similarity to sympathetic nervous activity. To a greater or lesser extent all these chemicals imitated sympathetic stimulation and Dale coined the word ‘sympathomimetic’ to describe them [33]. Intriguingly one such chemical, noradrenaline, provided the closest ‘match’ to nerve stimulation, but at the time noradrenaline was not known to occur naturally, and was simply not investigated further.

Another substance that was known only as a product of laboratory synthesis was acetylcholine. In 1913, however, a sample of ergot being tested at the WPRL by Dale was contaminated with a substance subsequently identified chemically as acetylcholine [34]. Dale tested it further using physiological techniques and wrote excitedly to Elliott:

“We got that thing out of our silly ergot extract. It is acetyl-choline and a most interesting substance. It is much more active than muscarine, though so easily hydrolysed that its action, when it is injected into the blood-stream, is remarkably evanescent, so that it can be given over & over again with exactly similar effects, like adrenaline. Here is a good candidate for the rôle of a hormone related to the rest of the autonomic nervous system. I am perilously near wild theorising” [35].

Similar to his study of sympathomimetic amines, Dale initiated an examination of the physiological effects of an array of choline derivatives, and noted two principal effects of acetylcholine: one that could be reproduced by injections of muscarine; and one reproduced by nicotine [36]. He offered no speculations to account for the functional differences, apart from commenting

“One may merely conclude that there is some degree of biochemical similarity between the ganglion cells of the whole involuntary system, and the terminations of voluntary nerve-fibres in striated muscle, on the one hand, and the mechanism connected with the peripheral termination of cranio-sacral involuntary nerves on the other….There does not seem to be the same relation between the affinities of ganglion cells and of the terminal mechanism connected with the true sympathetic system” [36].

But a major problem remained: acetylcholine had been found to occur naturally in only one sample of the fungus ergot. It had never been identified as a constituent of an animal body.

### Acceptance of chemical synaptic transmission: Henry Dale and Otto Loewi (1873–1961)

After the inevitable diversion of research effort during the First World War, work into possible chemical synaptic transmitters continued. In the 1920s, the Austrian pharmacologist Otto Loewi produced a demonstration that applied chemicals were responsible for the transmission of neural effects to the frog heart, especially a choline-like chemical that inhibited, and an adrenaline-like substance that accelerated, the heartbeat, although his interpretation was not immediately or widely accepted [37]. In 1927 Dale and Harold Dudley produced convincing evidence for the presence of acetylcholine in the animal body, and during the early 1930s Dale and his colleagues, especially G. L. Brown, Marthe Vogt and Wilhelm Feldberg, amassed considerable evidence for the role of acetylcholine as a neurotransmitter at synapses at autonomic ganglia, at the parasympathetic post-ganglionic junction and at the neuromuscular junction of the voluntary nervous system [38]. The concept of neurotransmission was also extended into the central nervous system, early interest in the possibility having been shown by Sherrington. In 1948 Dale recollected telling Sherrington over 20 years previously of all the evidence he and his colleagues had accumulated for acetylcholine as a neurotransmitter at pre-ganglionic and voluntary muscle synapses. Sherrington’s immediate response had been “to insist that a process observed at peripheral synapses must furnish some kind of analogy for events at those of the central nervous system” [39].

Dale however made a further, non-experimental but enormously significant contribution. In a Communication to the Physiological Society he proposed the use of two new words: ‘cholinergic’ and ‘adrenergic’ to designate nerve fibres by the nature of the chemical that they might use as a transmitter, rather than by any anatomical classification. The flexibility about a precise chemical identification allowed that neurotransmitters might be ‘adrenaline-like’ and ‘acetylcholine-like' but were not necessarily either of those two chemicals. As Dale’s final remark emphasises “I think such a usage would assist clear thinking, without committing us to precise chemical identifications, which may be long in coming” [40].

### Nobel Prizes: Sherrington and Adrian (1930), Dale and Loewi (1936), and beyond

The Nobel prizes, created by Alfred Nobel’s 1895 Will, were first awarded in 1901, to “those who, during the preceding year, have conferred the greatest benefit to Mankind” [41]. Five prizes were established in Peace, Literature, Chemistry, Physics, and Physiology or Medicine, the very title of the latter prize emphasising the dominant role that physiology and physiological sciences then commanded. The condition about ‘the preceding year’ was quickly disregarded, and over the years many Prizes have been awarded for studies on neural functioning and synaptic transmission, several to scientists mentioned in this account. The first such Prize was in 1932 to Charles Sherrington and Edgar Adrian “for their discoveries regarding the functions of neurons” and just four years later Henry Dale and Otto Loewi shared the award for “for their discoveries relating to chemical transmission of nerve impulses” [42].

Sometimes Nobel Prizes are awarded in respect of a whole body of well accepted work; at other times they appear to be conferred prematurely, before a consensus of scientific opinion and judgement has cohered in approval. The accolade for chemical neurotransmission was seemingly such an event, as the concept stimulated vigorous opposition from many prominent physiologists, most notably those from the ‘Sherrington school’ of neurophysiology, fronted by John Eccles (1903–1997). In a retrospective account, Eccles recalled how he had assessed the evidence for chemical transmission and while conceding that longer-acting (slower) transmission might be due to acetylcholine, short-acting (fast) transmission was clearly electrical, “thus it was supposed that transmission at neuro-muscular and neuronal synapses, which is many cases is accomplished within a few milliseconds, must be mediated by the electrical currents associated with the nerve impulse” [43]. It may however be pertinent to consider a private comment made by E. D. Adrian to Dale “I have been in the habit of sitting on any paper of Eccles for a year or so before letting myself be convinced by the apparently water-tight arguments” [44]. Debates about the roles of chemicals versus pure electrical transmission at the synapse, jocularly known as ‘spark versus soup’ and largely conducted at meetings of The Physiological Society, raged for several years [45].

Eccles' ultimate acceptance of chemical transmission resulted from a study of the effects of eserine, an anticholinesterase drug, on the endplate potential of a curarized muscle, in which the initial fast transmission, as well as the slow response, was due to acetylcholine [46]. The ensuing publication occasioned Eccles to write to Dale,

“I heard recently…that you and [G L] Brown were glad we had at last admitted the full significance of ACh at the neuro-muscular junction!” [47].

to which Dale replied

“I do not know from what source you may have heard of our reaction to your paper in the *Journal of Neurophysiology*, but the report which reached you was certainly not wholly inaccurate… I am told that John Fulton…has begun to balance himself more carefully than before on the top of the hedge, so that eventually we may find you all on the same, safe side” [47].

On the occasion of Dale’s ninetieth birthday in 1965, Eccles offered a pertinent tribute

“My memory takes me back more than three decades when Dale and his colleagues literally staggered us neurophysiologists by the hypothesis that even the fast synaptic transmissions were mediated chemically. After many years of resistance to this hypothesis, I came in 1951 to accept it unreservedly by what Sir Henry regards as the scientific equivalent of a religious conversion. I reciprocate appropriately by saying that he is one of my scientific saints” [48].

Eccles too was awarded a Nobel Prize in 1963, shared with Alan Hodgkin and Andrew Huxley “for their discoveries concerning the ionic mechanisms involved in excitation and inhibition in the peripheral and central portions of the nerve cell membrane” [42]. The theory of chemical neurotransmission was substantially strengthened by the discovery by Ulf von Euler in 1947 of noradrenaline as a naturally occurring substance, not purely the synthetic novelty that Dale and Barger had identified as being the most potent sympathomimetic in 1910 [33]. Euler duly also won a Nobel Prize, in 1970, shared with Bernard Katz and Julius Axelrod “for their discoveries concerning the humoral transmitters in the nerve terminals and the mechanism for their storage, release and inactivation.”[42]

In 1958, Dale wrote to his old friend and colleague Tom Elliott about “a happy morning” he had spent discussing possible mechanisms of cholinergic transmission with Katz, then Professor of Biophysics at University College London. Dale explained

“I feel almost bewildered by the kind of detail which such people are now elucidating with the aid of electron-ultramicroscopy, and also with an electrical recording which they can now achieve of the transmitted excitatory process at the motor end-plate of a single muscle fibre. … A great deal indeed has happened since you first suggested a chemical mechanism for the transmission of the excitatory process from a nerve ending and it goes on happening with a constant acceleration” [49].

## Data Availability

All original archives available as indicated.

## Abbreviations

GHL: George Henry Lewes
WPRL: Wellcome Physiological Research Laboratories
ACh: Acetylcholine
